# The Protective Effect of Qishen Granule on Heart Failure after Myocardial Infarction through Regulation of Calcium Homeostasis

**DOI:** 10.1155/2020/1868974

**Published:** 2020-10-22

**Authors:** Xiaomin Yang, Qiyan Wang, Zifan Zeng, Qian Zhang, Fang Liu, Hong Chang, Chun Li, Wei Wang, Yong Wang

**Affiliations:** ^1^School of Life Sciences, Beijing University of Chinese Medicine, Beijing 100029, China; ^2^State Key Laboratory of Bioactive Substances and Function of Natural Medicine, Institute of Materia Medica, Peking Union Medical College and Chinese Academy of Medical Sciences, Beijing 100050, China; ^3^College of Chinese Medicine, Beijing University of Chinese Medicine, Beijing 100029, China; ^4^School of Chinese Materia Medica, Beijing University of Chinese Medicine, Beijing 100029, China; ^5^Traditional Chinese Medicine College, North China University of Science and Technology, Tangshan, Hebei 063210, China; ^6^Modern Research Center for Traditional Chinese Medicine, School of Chinese Materia Medica, Beijing University of Chinese Medicine, Beijing 100029, China

## Abstract

Qishen granule (QSG) is a frequently prescribed traditional Chinese medicine formula, which improves heart function in patients with heart failure (HF). However, the cardioprotective mechanisms of QSG have not been fully understood. The current study aimed to elucidate whether the effect of QSG is mediated by ameliorating cytoplasmic calcium (Ca^2+^) overload in cardiomyocytes. The HF rat model was induced by left anterior descending (LAD) artery ligation surgery. Rats were randomly divided into sham, model, QSG-low dosage (QSG-L) treatment, QSG-high dosage (QSG-H) treatment, and positive drug (diltiazem) treatment groups. 28 days after surgery, cardiac functions were assessed by echocardiography. Levels of norepinephrine (NE) and angiotensin II (AngII) in the plasma were evaluated. Expressions of critical proteins in the calcium signaling pathway, including cell membrane calcium channel CaV1.2, sarcoendoplasmic reticulum ATPase 2a (SERCA2a), calcium/calmodulin-dependent protein kinase type II (CaMKII), and protein phosphatase calcineurin (CaN), were measured by Western blotting (WB) and immunohistochemistry (IHC). Echocardiography showed that left ventricular ejection fraction (EF) and fractional shortening (FS) value significantly decreased in the model group compared to the sham group, and illustrating heart function was severely impaired. Furthermore, levels of NE and AngII in the plasma were dramatically increased. Expressions of CaV1.2, CaMKII, and CaN in the cardiomyocytes were upregulated, and expressions of SERCA2a were downregulated in the model group. After treatment with QSG, both EF and FS values were increased. QSG significantly reduced levels of NE and AngII in the plasma. In particular, QSG prevented cytoplasmic Ca^2+^ overload by downregulating expression of CaV1.2 and upregulating expression of SERCA2a. Meanwhile, expressions of CaMKII and CaN were inhibited by QSG treatment. In conclusion, QSG could effectively promote heart function in HF rats by restoring cardiac Ca^2+^ homeostasis. These findings revealed novel therapeutic mechanisms of QSG and provided potential targets in the treatment of HF.

## 1. Introduction

Heart failure remains one of the major threats to people's health, although great progress has been made in the understanding of HF pathophysiology and advances in its therapeutic strategies [1]. It poses the entire medical community a tremendous challenge to further explore HF pathogenesis and the treatment approaches. In recent decades, the maintenance of cardiac calcium homeostasis in HF development has been extensively investigated due to its roles in HF progress [2, 3]. Of note, cardiac calcium overload is so essential that it is considered as the attractive target for treating HF. Therefore, the calcium channel blockers in the management of HF has gained a great interest worldwide [4, 5].

Changes of calcium concentration in cardiomyocytes determine the contractility of the heart, thus to influence the heart function. Free calcium ions are mainly distributed in the extracellular fluid such as blood and intracellular organelles such as sarcoplasmic reticulum. However, it is the cytosolic Ca^2+^ concentration that directly determines the myocardial contractility. During cardiac excitation-contraction coupling, calcium concentration in the cytosol increases approximately 10-fold because extracellular Ca^2+^ flows into the cardiomyocyte via the cardiac cell membrane calcium channel CaV1.2 and triggers more Ca^2+^ release from the sarcoplasmic reticulum membranes [6, 7]. Relaxation occurs when Ca^2+^ is pumped back into the SR by SERCA2a [8, 9]. In the failing heart, cytosolic calcium is overload due to excessive calcium entry from the extracellular fluid and SR or reduced calcium efflux from the cytosol. Abnormally activated CaV1.2 or reduced expression of SERCA2a leads to accumulation of Ca^2+^ in the cytosol, which prevents relaxation and further impairs contractility due to depletion of the Ca^2+^ available for release during systole [10, 11].

Furthermore, CaMKII and CaN in the cytosol are two of the most important calcium-dependent signaling proteins, which can bind with the increasing Ca^2+^ in the cytosol, thus to induce cardiac hypertrophy and remodeling directly [12–14]. Cytosolic calcium overload during HF could be induced by excessive activation of two core signaling pathways, including the *β*-adrenergic signaling pathway and renin-angiotensin-aldosterone pathway [15, 16]. Activation of the *β*-adrenergic pathway by NE activates the protein kinase A (PKA) and CaMKII and subsequently phosphorylates CaV1.2, which results in an increase of cytosolic calcium entry from the extracellular fluid [17]. Meanwhile, phosphorylation of the ryanodine receptor isoform 2 by PKA leads to diastolic leakage of calcium from the SR during heart failure [18]. Moreover, activation of AngII in HF can facilitate calcium flow into the myocardial cells by changing the permeability of CaV1.2, and increase cytosolic calcium ions by promoting the release of calcium from SR, which finally lead to myocardial fibrosis [19].

Traditional Chinese medicine (TCM) has been applied in the treatment and prevention of HF for thousands of years. Qishen granule, one of the most widely prescribed herbal formulae, is composed of six herbs, including *Radix Astragali Mongolici, Salvia miltiorrhiza Bunge, Flos Lonicerae, Scrophularia, Radix Aconiti Lateralis Preparata,* and *Radix Glycyrrhizae*. This formulae is widely manufactured in China in accordance with the China Pharmacopoeia standard of quality control. The fingerprint of QSG was analyzed by HPLC-IT-TOF-MS, and the typical chromatograms were reported as we described before [20]. Our previous studies have demonstrated that QSG has a definite effect in improving heart function. Study based on network pharmacology predicted that the calcium signaling pathway was one of the most potential drug targets of QSG [21]. However, whether its efficacy is related to amelioration of abnormal accumulation of Ca^2+^ in the cytosol remains poorly defined.

In this study, we aim to investigate the underlying pharmacological mechanisms of QSG in the HF model. The effects of QSG on calcium transfer proteins and calcium/calmodulin-dependent enzymes were studied. The HF model was induced by ligation of the left anterior descending coronary artery in rats. This study will provide insight into the therapeutic mechanisms of QSG and provide the experimental basis for its clinical application.

## 2. Materials and Methods

### 2.1. Herbs Preparation

QSG were composed of 460 g *Radix Astragali Mongolici*, 230 g *Salvia miltiorrhiza Bunge*, 160 g *Flos Lonicerae*, 160 g *Scrophularia*, 140 g *Radix Aconiti Lateralis Preparata*, and 90 g *Radix Glycyrrhizae*. These herbs were purchased from Beijing Tong Ren Tang Chinese Medicine Co., Ltd. (Beijing, China) and prepared in the traditional Chinese medicine preparation department of Beijing China-Japan Friendship Hospital. The major extraction steps were performed as described previously [20]. Briefly, herbs were extracted by water for three times. Then, the water extract was concentrated and precipitated by ethanol. The sediment was dried and screened over an 80-mesh sieve for crushing. After preparation, the extracted QSG was enriched by 4 times. The fingerprint spectrum was further established by the high-performance liquid chromatography (HPLC) method in our previous studies [20, 22]. The major components are formononetin, tanshinone IIA, tanshinone I, cryptotanshinone, and harpagoside [23]. The Chinese herbs were identified by Professor Jian Ni, School of Chinese Materia Medica, Beijing University of Chinese Medicine. The voucher specimens (Voucher numbers: HQ-2016-007; DQ-2016-008; JYH-2016-009; XS-2016-010; FZ-2016-011; and GC-2016-012) were submitted to Department of Chinese Medicine Teaching and Research, School of Traditional Chinese Medicine, Beijing University of Chinese Medicine.

### 2.2. Animal Grouping and Induction of Acute Myocardial Infarction (AMI)

Sixty male Sprague-Dawley (SD) rats (weighted 220 ± 10 g) were randomly divided into five groups including sham-operated, model, QSG-low dosage treatment, QSG-high dosage treatment, and positive drug (diltiazem) treatment groups. Rats were purchased from Beijing Si Bei Fu Biotechnology Co., Ltd. (Beijing, China). Studies were performed with the approval of the Animal Care Committee of Beijing University of Chinese Medicine. The rats were regularly fed for one week before surgery.

Models of HF after AMI were induced by left anterior descending artery ligation surgeries. The operational procedure has been described in our previous study [21]. Briefly, left thoracotomies of the rats were performed after anaesthetized via intraperitoneal injection by 1% pentobarbital sodium at the dosage of 50 mg/kg. LAD coronary arteries were then ligated with 5-0 polypropylene sutures. After ligation, the thoraxes were closed, and the rats were given sodium penicillin for 3 continuous days to prevent the potential inflammation. Rats in the sham group received the same procedure except that the coronary arteries were not ligated. A total of 25 rats died within 24 hrs after the surgery due to lethal arrhythmias or acute pump failure. 7 rats in each group were reserved for further research, and they were all analyzed at the end of study. From the second day after surgery, rats were treated with drugs or water via oral gavage for 28 consecutive days. QSG-L and QSG-H treatment groups received QSG at the raw dosage of 9.33 g/kg or 18.66 g/kg per day, respectively. The positive group received diltiazem at the dosage of 37.5 mg/kg per day. QSG and diltiazem were dissolved in water. Animals in the sham and model groups were given a gavage of normal water. After 28 days, cardiac functions were assessed by echocardiography. The rats were anaesthetized using 1% pentobarbital sodium at the dosage of 50 mg/kg via intraperitoneal injection, then blood samples were collected through abdominal aorta puncture, and hearts were harvested. The tissues were stored in the liquid nitrogen or 4% paraformaldehyde for further use.

### 2.3. Measurement of Cardiac Functions

Cardiac functions were assessed by echocardiography after rats were anaesthetized at 28 days after surgery. By using a Vevo 2100 (Visual Sonics Inc, Toronto, Ontario, Canada) and a PST 65A sector scanner (8 MHz probe), two dimensional images were generated at a frame rate of 300–500 frames/s. The parameters of cardiac functions include left ventricular ejection fraction, fractional shortening, left ventricular internal dimension-systole (LVID; s), and left ventricular internal dimension-diastole (LVID; d).

### 2.4. Morphometric Analysis

The new harvested hearts were crosscut 5 mm below the ligature. The upper parts of hearts were fixed in 4% paraformaldehyde for 72 hrs. After that, heart tissues were embedded in paraffin and sectioned into 5 *μ*m slices. Masson's staining was applied to evaluate the degree of myocardial fibrosis.

### 2.5. Measurement of Plasma Indicators

Level of norepinephrine in plasma was measured by enzyme-linked immunosorbent assay (ELISA) (ST-360 microplate reader, Shanghai Ke Hua Co., Ltd., Shanghai, China). Blood was left at room temperature for 30 min and then centrifuged for 10 min at 3000*g*. The upper plasma was collected for measurement. Level of angiotensin II in plasma was measured by radioimmunoassay (RIA) (DFM-96 radioimmunoanalyzer, Hefei Zhong Cheng electromechanical Co., Ltd., Hefei, China). The blood was homogenized on ice in saline containing an enzyme inhibitor (0.30 M EDTA-Na 10 *μ*L, 0.34 M 8-hydroxyquinoline 10 *μ*L, and 0.32 M dimercaptopropanol 5 *μ*L per mL blood). The homogenate was centrifuged at 3000*g* for 10 min, and then, the supernatant was used for determination. Norepinephrine and angiotensin II were both determined in Beijing Zhong Tong Lan Bo Clinical Laboratory (Beijing, China). Each assay was performed following respective instructions. Standards at a series of concentrations were run in parallel with the samples. The concentrations in the samples were calculated in reference to the corresponding standard curves.

### 2.6. Measurement of Indicators by Western Blotting

Cardiac tissues were lysed using RIPA buffer (50 mM Tris-HCl pH 7.4, 150 mM NaCl, 1% NP-40, and 0.1%SDS) containing a protease inhibitor cocktail. Total proteins were extracted from these homogenates, and protein concentrations were measured by a protein assay kit. After boiling for 5 min, equal amounts of protein extracts (50 *μ*g) were separated by 10% sodium dodecyl sulphate- (SDS-) polyacrylamide gel electrophoresis (Bio-Rad, CA, U.S.A.) and transferred onto PVDF membranes electrophoretically. The membranes then were blocked with 5% nonfat dry milk in Tris buffered saline (20 mM Tris, pH 7.6, and 137 mM NaCl) with 0.1% Tween 20 followed by incubation with primary antibodies. Primary antibodies employed included mouse monoclonal anticalcium channel L type DHPR alpha 2 subunit (CaV1.2, ab2864, Abcam, UK), rabbit monoclonal anti-SERCA2 ATPase (SERCA2a, ab137020, Abcam, UK), rabbit monoclonal anti-CaMKII delta (CaMKII, ab181052, Abcam, UK), rabbit monoclonal anticalcineurin A (CaN, ab52761, Abcam, UK), and mouse monoclonal antiglyceraldehyde-3-phosphate dehydrogenase (GAPDH, ab8245, Abcam, UK). After incubation with the primary antibodies, the membranes were washed for 3 times, and then incubated with the secondary antibodies (SA00001-1 or SA00001-2, Proteintech Group, Inc., U.S.A.). The membranes were treated with ECL Plus Western blotting detection reagent for 1 min at room temperature. The bands in the membrane were visualized and analyzed using UVP BioImaging Systems. Protein expressions were normalized by the GAPDH band densities.

### 2.7. Measurement of Indicators by IHC

IHC was performed by using immunohistochemistry kits (Wuhan Service Biotechnology Co., Ltd., Wuhan, China). Briefly, histological sections were deparaffinized in xylene, rehydrated in alcohol gradient, and then rinsed in water. Following antigen retrieval, the sections were treated with 3% hydrogen peroxide to block endogenous peroxides and incubated with 3% bovine serum albumin to block nonspecific staining. The slides were then incubated with primary antibody (mouse monoclonal anticalcium channel L type DHPR alpha-2 subunit, CaV1.2, ab2864, Abcam, UK) overnight at 4°C. The immunoreaction was achieved with the secondary antibody (the goat antimouse antibody, SA00001-1, Proteintech Group, Inc., U.S.A.) and developed with 3,3′-diaminobenzidine tetrahydrochloride (DAB). After they were stained by hematoxylin for 3 min, the slides were dehydrated by graded ethanol and xylene and mounted with rhamsan gum. The immunostaining results were observed under the optical microscope and photographed. Positive area was specific stained brown–yellow. The proportion of positive area to total tissue area represented CaV1.2 expressions.

### 2.8. Statistical Analysis

Statistical analyses were performed by the one-way analysis of variance (ANOVA) test using SPSS 17.0 software. *P* < 0.05 was considered statistically significant. Data were presented as mean ± standard deviation (mean ± SD).

## 3. Results

### 3.1. QSG Could Restore Heart Function and Ameliorate Myocardial Fibrosis in HF Rats after AMI

**Figure 1:**
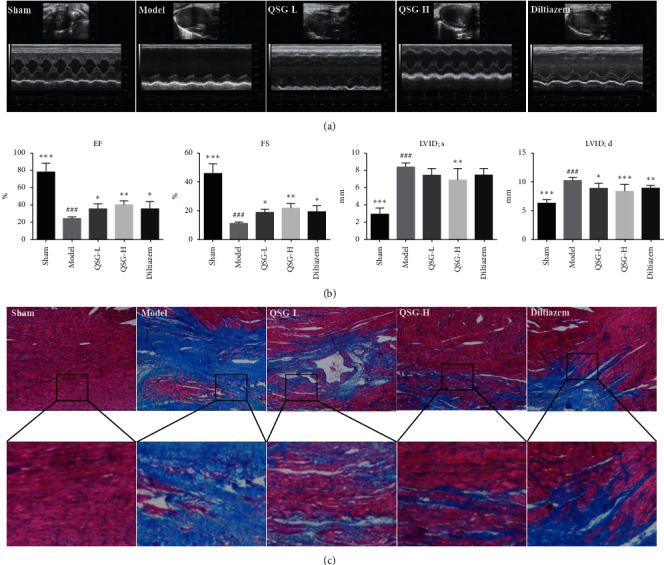
QSG could restore heart function and ameliorate myocardial fibrosis in HF rats after AMI. (a) Representative images of 2D echocardiogram in sham, model, QSG-L, QSG-H, and diltiazem groups. (b) Echocardiographic measurements of EF, FS, LVID; s, and LVID; d. QSG-L and QSG-H improved left ventricular EF and FS. (c) Masson staining indicated that QSG-L and QSG-H preserved cardiomyocyte structure and inhibited myocardial fibrosis. ^##^*P* < 0.01 and ^###^*P* < 0.001 vs. the sham group; ^*∗*^*P* < 0.05, ^*∗∗*^*P* < 0.01, and ^*∗∗∗*^*P* < 0.001 vs. the model group.

Parameters of cardiac functions were detected by echocardiography. Representative *M*-mode frames are shown in Figure 1(a). Values of EF and FS in the model group decreased by 68.90% (*P* < 0.001) and 74.63% (*P* < 0.001) compared with those in the sham group. Meanwhile, LVID; s and LVID; d in the model group increased significantly by 185.03% (*P* < 0.001) and 61.13% (*P* < 0.001) as compared with those in the sham group. EF and FS values in the QSG-L group were upregulated by 44.47% (*P* < 0.05) and 65.17% (*P* < 0.05), respectively, compared with those in the model group. In the QSG-H group, EF and FS values were upregulated by 67.32% (*P* < 0.01) and 65.17% (*P* < 0.01). Diltiazem treatment also improved the function and ventricular structure (Figure 1(b)). The values of EF, FS, LVID; s, and LVID; d, are shown in Table 1.

Masson staining was applied to assess the degree of myocardial fibrosis. As shown in Figure 1(c), there was little interstitial collagen deposition in the sham group. However, extensive collagen deposition, as indicated by blue stains, was observed in the model group. In the QSG-L, QSG-H, and diltiazem groups, collagen deposition was inhibited as compared with the model group.

### 3.2. Effects of QSG on Plasma Indicators of NE and AngII in HF Rats after AMI

**Figure 2:**
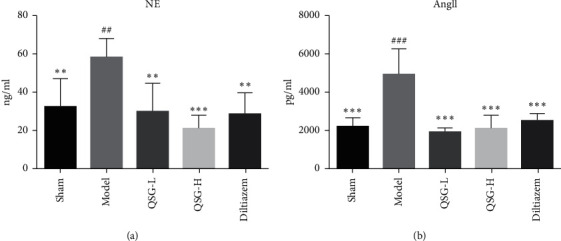
Effects of QSG on plasma indicators NE and AngII in HF rats after AMI. Plasma levels of NE (a) and AngII (b) in the five groups of rats were detected, respectively, by ELISA and RIA. QSG-L and QSG-H could decrease NE and AngII levels compared with the model group. Diltiazem had an effect similar to QSG-L and QSG-H groups. ^##^*P* < 0.01 and ^###^*P* < 0.001 vs. the sham group; ^*∗∗*^*P* < 0.01, ^*∗∗∗*^*P* < 0.001 vs. the model group.

The increase of plasma indicators NE and AngII could reflect the alteration of cardiac functions and influence the calcium level in cardiac myocytes. Compared with the sham group, the levels of NE (Figure 2(a)) and AngII (Figure 2(b)) of the model group were increased by 79.76% (*P* < 0.01) and 55.43% (*P* < 0.001), respectively. After treatment with QSG-L, the plasma levels of NE and AngII were decreased by 48.76% (*P* < 0.01) and 62.86% (*P* < 0.001), respectively, as compared with the model group. QSG-H treatment decreased NE and AngII by 60.70% (*P* < 0.001) and 58.13% (*P* < 0.001). The positive drug diltiazem also downregulated the plasma levels of NE and AngII. These results suggested that QSG had a cardioprotective effect under ischemic stimulus and had the potential role of reducing the calcium level in cardiac myocytes.

### 3.3. Effects of QSG on Expressions of CaV1.2 and SERCA2a in HF Rats after AMI

**Figure 3:**
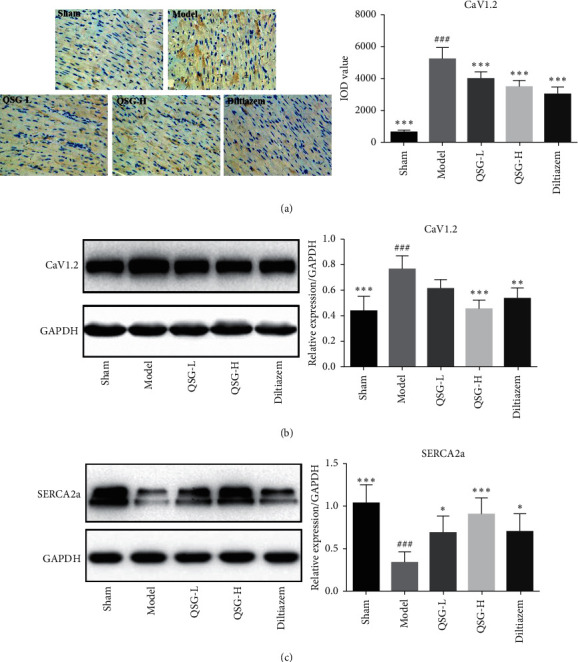
Effects of QSG on expressions of CaV1.2 and SERCA2a in HF rats after AMI. (a) Immunohistochemistry images of CaV1.2 and quantitative results in the heart tissues of rats in different groups. (b) WB bands of CaV1.2 and its quantitative results in the heart tissues of rats. (c) WB bands of SERCA2a and its quantitative results in the heart tissues of rats. IHC and WB results showed that the expression of CaV1.2 in the model group was upregulated compared with the sham group. QSG-L and QSG-H could decrease levels of CaV1.2 compared with the model group. Western blot showed that the expression of SERCA2a in the model group was downregulated compared with the sham group. QSG-L and QSG-H could increase protein levels of SERCA2a significantly compared with the model group. ^###^*P* < 0.001 vs. the sham group; ^*∗*^*P* < 0.05, ^*∗∗*^*P* < 0.01, and ^*∗∗∗*^*P* < 0.001 vs. the model group.

CaV1.2 was assessed by immunohistochemistry and Western blot. Integrated optical density (IOD) and expression of CaV1.2 in the model group were increased by 86.87% (*P* < 0.001) and 43.61% (*P* < 0.001) compared with the sham group. After treatment with QSG-L and QSG-H, the IOD of CaV1.2 were decreased by 23.34% (*P* < 0.001) and 33.46% (*P* < 0.001) respectively, and the protein levels were decreased by 20.41% (*P* > 0.05) and 41.19% (*P* < 0.001), respectively, indicating that QSG-L and QSG-H could reduce the flow of extracellular calcium into the cytoplasm of cardiac myocytes. The positive control drug diltiazem also significantly suppressed the expression of CaV1.2 (Figures 3(a) and 3(b)).

Expression of SERCA2a in the model group was downregulated significantly by 202.74% (*P* < 0.001) compared with the sham group. Treatment with QSG-L and QSG-H upregulated expressions of SERCA2a by 101.09% (*P* < 0.05) and 163.83% (*P* < 0.001), respectively, as compared with the model group. In diltiazem treatment group, the expression of SERCA2a was upregulated by 104.98% (*P* < 0.05) compared with that in the model group (Figure 3(c)).

### 3.4. Effects of QSG on Expressions of CaMKII and CaN in HF Rats after AMI

**Figure 4:**
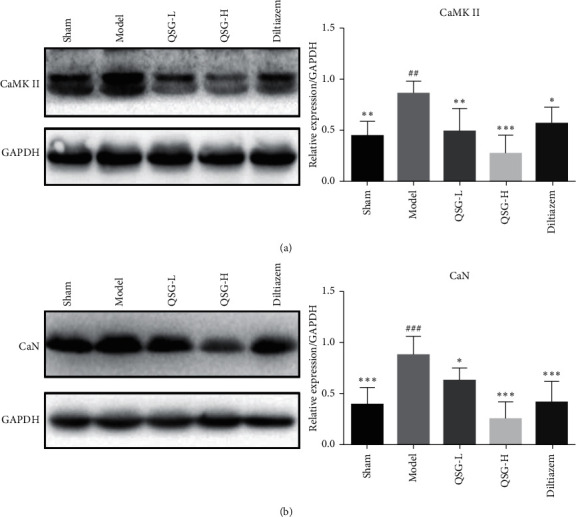
Effects of QSG on expressions of CaMKII and CaN in HF rats after AMI. (a) WB bands of CaMKII and its quantitative results in heart tissues of rats. (b) WB bands of CaN and its quantitative results in heart tissues of rats. Western blot showed that the expressions of CaMKII and CaN in the model group were upregulated compared with the sham group. QSG-L and QSG-H could decrease levels of CaMKII and CaN compared with the model group. ^##^*P* < 0.01 and ^###^*P* < 0.001 vs. the sham group; ^*∗*^*P* < 0.05, ^*∗∗*^*P* < 0.01, and ^*∗∗∗*^*P* < 0.001 vs. the model group.

To further confirm the effect of QSG on the calcium signaling pathway, levels of CaMKII and CaN were determined. Expressions of CaMKII and CaN were evaluated by WB. The protein level of CaMKII in the model group was upregulated by 48.86% (*P* < 0.01) compared with the sham group. In QSG-L and QSG-H group, the expressions of CaMKII were downregulated by 44.50% (*P* < 0.01) and 68.16% (*P* < 0.001), respectively, compared with the model group. Diltiazem also downregulated the expression of CaMKII (*P* < 0.05), but the effect of diltiazem on CaMKII was milder than that of QSG-L and QSG-H groups (Figure 4(a)). Expression of CaN in the model group was increased by 55.33% (*P* < 0.001) compared with the sham group. Treatment with QSG-L and QSG-H downregulated expressions of CaN by 29.88% (*P* < 0.05) and 71.34% (*P* < 0.001), respectively, as compared with the model group. In diltiazem treatment group, expression of CaN was downregulated by 53.16% (*P* < 0.001) compared with those in the model group (Figure 4(b)).

## 4. Discussion

Traditional Chinese medicine has been widely used in the prevention and treatment of HF for thousands of years. QSG is a patent formula of TCM and has been shown to be effective in treating HF [24, 25]. In this study, by applying the rat model of heart failure after myocardial infarction, we investigated the pharmacological mechanisms of QSG in the treatment of myocardial infarction. Our major findings are as follows: (1) QSG could effectively improve the cardiac functions and ameliorate myocardial fibrosis in rats after AMI. (2) The therapeutic effects of QSG may be mediated by ameliorating cytoplasmic Ca^2+^ overload in the cardiac cells, manifested by downregulation of cell membrane calcium channel CaV1.2 and upregulation of the SERCA2a. (3) CaMKII and CaN were inhibited by QSG treatment, which provided further evidence that QSG protected cardiac functions through the calcium signaling pathway.

Calcium signaling pathway plays a critical role in the process of heart failure. Calcium influx is also considered to account for many detrimental effects of traditional inotropic drugs, such as glycosides digoxin [26]. Imbalance of calcium homeostasis in the cardiomyocytes not only directly interferes myocardial contraction and relaxation through calcium/calmodulin-dependent proteins but also exacerbates the heart failure process by interacting with other pathophysiological factors such as the *β*-adrenergic signaling pathway and renin-angiotensin-aldosterone system (RAAS) [27–30]. These pathophysiological changes were also confirmed by our experimental results. Heart function and the structure in HF rats after AMI were severely impaired, as illustrated by decreased EF and FS values and increased degrees of myocardial fibrosis in the model group compared to the sham group. In addition, expressions of critical channel proteins in the calcium signaling pathway were altered. Expression of CaV1.2 was upregulated, while SERCA2a was downregulated, and the levels of NE and AngII in the plasma were increased. These changes could inevitably lead to cytosolic Ca^2+^ overload by promoting extracellular Ca^2+^ influx and reducing the flow of cytoplasmic Ca^2+^ back into SR. Furthermore, Ca^2+^ overload in the cytosol would abnormally activate calcium/calmodulin-dependent protein kinase and protein phosphatase or upregulate their expressions. Our results showed that the expressions of CaMKII and CaN were definitely upregulated in the model group compared to the sham group. Our results were consistent with other studies which reported that the pathological changes of these molecules were found in HF. For example, it was reported that phosphorylation of CaV1.2 was increased during heart failure, and altered expression or mutation of CaV1.2 channels at Ser1700 caused reduced contractile function, cardiac hypertrophy, and heart failure [31, 32]. Furthermore, microdomain-targeted remodeling of CaV1.2 properties could influence calcium homeostasis and might contribute to ventricular arrhythmogenesis in the settings of HF-associated remodeling [33]. During heart failure, the expression of cardiac SERCA2a was downregulated, and the activity of SERCA2a was inhibited by its modulator, phospholamban [11]. It has been demonstrated that CaMKII *δ* contributes to mitochondrial dysfunction and the transition from hypertrophy to HF [34]. In addition to inducing HF, CaMKII could further aggravate cytoplasmic Ca^2+^ overload by phosphorylating CaV1.2 at Ser1512/Ser1570 and ryanodine receptor 2 (RyR2) at Ser2030/Ser2814 [35, 36]. CaN dephosphorylates and induces the translocation of cytoplasmic NFAT to the nucleus and subsequently activates the transcription of prohypertrophic target genes [37]. Studies have showed that the responses of calcineurin and its substrates depend upon its composition of specific subcellular domains [38]. During heart failure, elevated levels of NE in the blood activated PKA and lead to hyperphosphorylation of CaV1.2, which further enhanced Ca^2+^ induced Ca^2+^ release through CaV1.2 and RyR2, and resulted in a significant decrease in SERCA2a protein and activity [39, 40]. In addition, AngII was shown to reduce calcium transient amplitudes and cardiomyocyte contractile function in a rat model of HF [41]. AngII was also found to promote CaN-*β*-mediated calcium influx in cardiomyocytes, which resulted in the upregulation of the expression of ANP and cardiac hypertrophy [42].

**Figure 5:**
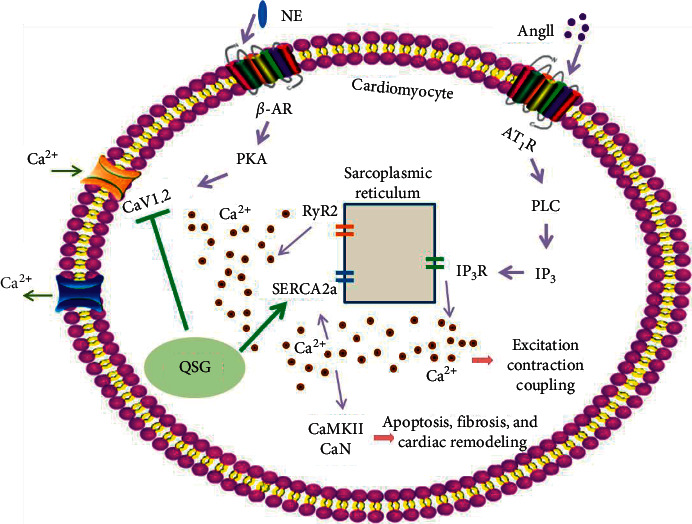
Potential mechanism of QSG efficacy on HF rats. QSG improved heart function and decreased cardiac remodeling by regulating the calcium signaling pathway. QSG ameliorated cytoplasmic Ca^2+^ overload in the cardiac cells, manifested by downregulation of CaV1.2 and upregulation of the SERCA2a and further confirmed by inhibition of CaMKII and CaN after QSG treatment.

In recent decades, calcium-handling proteins become the attractive targets of drugs research and development in HF therapies. For example, vepoloxamer, a rheologic agent, improved left ventricular function in dogs with advanced heart failure by inhibiting Ca^2+^ entry into cardiomyocytes [43]. Zacopride, an inward rectifier potassium channel agonist, alleviated cardiac hypertrophy and failure via alterations in calcium dyshomeostasis in rats [44]. As shown in our results, QSG significantly improved the EF and FS value and ameliorated myocardial fibrosis in HF rats after AMI. Moreover, CaMKII and CaN, the critical enzymes activated by high Ca^2+^ concentrations in the cytoplasm, were inhibited by QSG treatment. Studies have showed that inhibition of CaMKII suppressed diastolic Ca^2+^ waves, thus improving cardiac function in isoproterenol-challenged HF myocytes [45]. Excessive and sustained activation of CaN in the heart leads to fundamental changes in a number of substrates that often act in a feed-forward fashion, which contributes to pathological hypertrophic remodeling and accelerates cardiac decline [38]. In addition to QSG, several other traditional medicines were also found to act by inhibiting activities of CaMKII and CaN. For example, the YiQiFuMai powder injection significantly attenuated HF and improved the cardiac function by downregulating phosphorylation of CaMKII [46]. Buckwheat rutin exhibited an inhibitory effect on AngII-induced hypertrophy in cultured neonatal rat cardiomyocytes via Ca^2+^ antagonism action, thus blocking the CaN-dependent signal pathway [47]. Futhermore, our results showed that QSG downregulated CaV1.2 and upregulated SERCA2a, which would reduce the excessive extracellular calcium influx and promote the cytosolic calcium back into the SR, thus partly preventing cytosolic Ca^2+^ overload in the cardiac cells. Levels of NE and AngII in the plasma decreased in the QSG group compared with the model group, which was beneficial to ameliorate cytosolic Ca^2+^ overload, as chronic activation of the sympathetic nervous system and RAAS are thought to be main reasons of defective Ca^2+^ handling in failing hearts. The pharmacological mechanisms of QSG in the treatment of HF might be mediated by downregulating CaV1.2 and upregulating SERCA2a, as shown in Figure 5. Similar with our findings, many studies have shown that therapies targeting the abovementioned critical molecules in the calcium signaling pathway were beneficial for treating HF [48–51]. Inhibiton of L-type calcium channel reversed the susceptibility of atrial fibrillation in isoproterenol-induced HF mouse [48]. Gene-based therapies targeting SERCA2a led to improvements in calcium homeostasis and excitation-contraction coupling [50]. A novel pyrimidine-based CaMKII inhibitor was found to have the ability of increasing SR Ca^2+^ accumulation, and thus improving cardiomyocyte function effectively [49]. CaN inhibition in mice was shown to attenuate pathological cardiac hypertrophy [51].

## 5. Conclusions

QSG has regulatory effects on the calcium signaling pathway in cardiomyocytes of rats with heart failure, and the effects are mainly mediated by activating SERCA2a and inhibiting CaV1.2, CaMKII, and CaN. This study provides further insight into the therapeutic mechanism of QSG and proposes new strategies in the management of cardiovascular diseases by TCM.

## Figures and Tables

**Table 1:** Indicators of heart functions tested by echocardiography in different groups of rats.

Group	Sham	Model	QSG-L	QSG-H	Diltiazem
EF (%)	78.726 ± 9.228	24.483 ± 1.787	35.372 ± 5.861	40.965 ± 3.756	36.075 ± 7.323
FS (%)	46.033 ± 5.735	11.640 ± 0.691	19.497 ± 1.681	21.579 ± 2.976	19.576 ± 3.149
LVID; d (mm)	2.983 ± 0.628	8.502 ± 0.362	7.552 ± 0.599	6.895 ± 1.147	7.517 ± 0.702
LVID; d (mm)	6.432 ± 0.457	10.364 ± 0.394	9.014 ± 0.759	8.493 ± 1.050	8.943 ± 0.373

Data are presented as mean ± standard deviation (mean ± SD).

## Data Availability

The datasets used and analyzed during this study are available from the corresponding author upon reasonable request.

## References

[1] Virani S. S., Alonso A., Benjamin E. J. (2020). Heart disease and stroke statistics-2020 update: a report from the American heart association. *Circulation*.

[2] Djousse P. (2019). Noncoding RNAs in cardiovascular diseases. *Current Opinion in Cardiology*.

[3] Yampolsky P., Koenen M., Mosqueira M. (2019). Augmentation of myocardial I(f) dysregulates calcium homeostasis and causes adverse cardiac remodeling. *Nature Communications*.

[4] Mahon N., McKenna W. J. (1998). Calcium channel blockers in cardiac failure. *Progress in Cardiovascular Diseases*.

[5] Lam C. K., Tian L., Belbachir N. (2019). Identifying the transcriptome signatures of calcium channel blockers in human induced pluripotent stem cell-derived cardiomyocytes. *Circulation Research*.

[6] Bers D. M. (2014). Cardiac sarcoplasmic reticulum calcium leak: basis and roles in cardiac dysfunction. *Annual Review of Physiology*.

[7] Sato D., Dixon R. E., Santana L. F., Navedo M. F. (2018). A model for cooperative gating of L-type Ca^2+^ channels and its effects on cardiac alternans dynamics. *PLoS Computational Biology*.

[8] Jeong D., Yoo J., Lee P. (2018). miR-25 tough decoy enhances cardiac function in heart failure. *Molecular Therapy*.

[9] Quan C., Li M., Du Q. (2019). SPEG controls calcium reuptake into the sarcoplasmic reticulum through regulating SERCA2a by its second kinase-domain. *Circulation Research*.

[10] Ouyang J., Cao J., Jiang X., Xu L., Wang Y. (2017). Kv4.3 expression reverses ICa remodeling in ventricular myocytes of heart failure. *Oncotarget*.

[11] Zhang Y., Jiao L., Sun L. (2018). LncRNA ZFAS1 as a SERCA2a inhibitor to cause intracellular Ca^2+^ overload and contractile dysfunction in a mouse model of myocardial infarction. *Circulation Research*.

[12] Pan K., Chen H. (2019). MiR-625-5p inhibits cardiac hypertrophy through targeting STAT3 and CaMKII. *Human Gene Therapy Clinical Development*.

[13] Tenma T., Mitsuyama H., Watanabe M. (2018). Small-conductance Ca^2+^-activated K^+^ channel activation deteriorates hypoxic ventricular arrhythmias via CaMKII in cardiac hypertrophy. *American Journal of Physiology-Heart and Circulatory Physiology*.

[14] Tsutsui S., Lozano-Vidal N., López-Maderuelo M. D., Jiménez-Borreguero L. J., Armesilla Á. L., Redondo J. M. (2019). Cardiomyocyte calcineurin is required for the onset and progression of cardiac hypertrophy and fibrosis in adult mice. *The FEBS Journal*.

[15] Kamada R., Yokoshiki H., Mitsuyama H. (2019). Arrhythmogenic *β*-adrenergic signaling in cardiac hypertrophy: the role of small-conductance calcium-activated potassium channels via activation of CaMKII. *European Journal of Pharmacology*.

[16] Soares D. d. S., Pinto G. H., Lopes A. (2019). Cardiac hypertrophy in mice submitted to a swimming protocol: influence of training volume and intensity on myocardial renin-angiotensin system. *American Journal of Physiology-Regulatory, Integrative and Comparative Physiology*.

[17] Yu H., Yuan C., Westenbroek R. E., Catterall W. A. (2018). The AKAP Cypher/Zasp contributes to *β*-adrenergic/PKA stimulation of cardiac CaV1.2 calcium channels. *Journal of General Physiology*.

[18] Marx S. O., Reiken S., Hisamatsu Y. (2000). PKA phosphorylation dissociates FKBP12.6 from the calcium release channel (ryanodine receptor): defective regulation in failing hearts. *Cell*.

[19] Mathieu S., El Khoury N., Rivard K., Paradis P., Nemer M., Fiset C. (2018). Angiotensin II overstimulation leads to an increased susceptibility to dilated cardiomyopathy and higher mortality in female mice. *Scientific Reports*.

[20] Xia K., Wang Q., Li C., Zeng Z., Wang Y., Wang W. (2017). Effect of QSKL on MAPK and RhoA pathways in a rat model of heart failure. *Evidence-Based Complementary and Alternative Medicine*.

[21] Wang Y., Liu Z., Li C. (2012). Drug target prediction based on the herbs components: the study on the multitargets pharmacological mechanism of qishenkeli acting on the coronary heart disease. *Evidence-Based Complementary and Alternative Medicine*.

[22] Wang Y., Lin W., Li C. (2017). Multipronged therapeutic effects of Chinese herbal medicine qishenyiqi in the treatment of acute myocardial infarction. *Frontiers in Pharmacology*.

[23] Zhang Q., Shi J., Guo D. (2020). Qishen Granule alleviates endoplasmic reticulum stress-induced myocardial apoptosis through IRE-1-CRYAB pathway in myocardial ischemia. *Journal of Ethnopharmacology*.

[24] Li Y., Wang S., Zhao Z. (2013). Clinical assessment of complementary treatment with Qishen Yiqi dripping pills on ischemic heart failure: study protocol for a randomized, double-blind, multicenter, placebo-controlled trial (CACT-IHF). *Trials*.

[25] Huang R., Cui Y.-C., Wei X.-H. (2019). A novel traditional Chinese medicine ameliorates fatigue-induced cardiac hypertrophy and dysfunction via regulation of energy metabolism. *American Journal of Physiology-Heart and Circulatory Physiology*.

[26] Cummings E. D., Swoboda H. D. (2020). *Digoxin Toxicity*.

[27] Luo M., Anderson M. E. (2013). Mechanisms of altered Ca^2+^ handling in heart failure. *Circulation Research*.

[28] Mora M. T., Ferrero J. M., Romero L., Trenor B. (2017). Sensitivity analysis revealing the effect of modulating ionic mechanisms on calcium dynamics in simulated human heart failure. *PLoS One*.

[29] Babick A., Chapman D., Zieroth S., Elimban V., Dhalla N. S. (2012). Reversal of subcellular remodelling by losartan in heart failure due to myocardial infarction. *Journal of Cellular and Molecular Medicine*.

[30] Fowler E. D., Drinkhill M. J., Norman R. (2018). Beta1-adrenoceptor antagonist, metoprolol attenuates cardiac myocyte Ca^2+^ handling dysfunction in rats with pulmonary artery hypertension. *Journal of Molecular and Cellular Cardiology*.

[31] Yang L., Dai D.-F., Yuan C. (2016). Loss of *β*-adrenergic-stimulated phosphorylation of CaV1.2 channels on Ser1700 leads to heart failure. *Proceedings of the National Academy of Sciences*.

[32] Koval O. M., Guan X., Wu Y. (2010). C aV1.2 beta‐subunit coordinates CaMKII-triggered cardiomyocyte death and afterdepolarizations. *Proceedings of the National Academy of Sciences*.

[33] Hund J. L., Bhargava A., O’Hara T. (2016). Microdomain-specific modulation of L-type calcium channels leads to triggered ventricular arrhythmia in heart failure. *Circulation Research*.

[34] Punjabi B. D., Ling H., Divakaruni A. S. (2015). Mitochondrial reprogramming induced by CaMKII*δ* mediates hypertrophy decompensation. *Circulation Research*.

[35] Miyamoto A., Welling A., Fischer S. (2010). Facilitation of murine cardiac L-type Cav1.2 channel is modulated by calmodulin kinase II-dependent phosphorylation of S1512 and S1570. *Proceedings of the National Academy of Sciences*.

[36] Belevych A. E., Sansom S. E., Terentyeva R. (2011). MicroRNA-1 and -133 increase arrhythmogenesis in heart failure by dissociating phosphatase activity from RyR2 complex. *PLoS One*.

[37] Bueno O. F., van Rooij E., Molkentin J. D., Doevendans P. A., De Windt L. J. (2002). Calcineurin and hypertrophic heart disease: novel insights and remaining questions. *Cardiovascular Research*.

[38] Parra V., Rothermel B. A. (2017). Calcineurin signaling in the heart: the importance of time and place. *Journal of Molecular and Cellular Cardiology*.

[39] Ryall J. G., Schertzer J. D., Murphy K. T., Allen A. M., Lynch G. S. (2008). Chronic *β*2-adrenoceptor stimulation impairs cardiac relaxation via reduced SR Ca^2+^-ATPase protein and activity. *American Journal of Physiology-Heart and Circulatory Physiology*.

[40] Lei M., Wang X., Ke Y., Solaro R. J. (2015). Regulation of Ca(2+) transient by PP2A in normal and failing heart. *Frontiers in Physiology*.

[41] Hohendanner F., Bode D., Primessnig U. (2018). Cellular mechanisms of metabolic syndrome-related atrial decompensation in a rat model of HFpEF. *Journal of Molecular and Cellular Cardiology*.

[42] Pieske Y., Zhou Y., Cao Z. (2016). miR-155 functions downstream of angiotensin II receptor subtype 1 and calcineurin to regulate cardiac hypertrophy. *Experimental and Therapeutic Medicine*.

[43] Sabbah H. N., Zhang K., Gupta R. C., Emanuele M. (2020). Effects of intravenous infusion of vepoloxamer on left ventricular function in dogs with advanced heart failure. *Cardiovascular Drugs and Therapy*.

[44] Liu Q. H., Qiao X., Zhang L. J. (2019). I(K1) channel agonist zacopride alleviates cardiac hypertrophy and failure via alterations in calcium dyshomeostasis and electrical remodeling in rats. *Frontiers in Pharmacology*.

[45] Belevych A. E., Ho H. T., Bonilla I. M. (2017). The role of spatial organization of Ca^2+^ release sites in the generation of arrhythmogenic diastolic Ca^2+^ release in myocytes from failing hearts. *Basic Research in Cardiology*.

[46] Zhang Y., Zhang L., Zhang Y. (2019). YiQiFuMai powder injection attenuates coronary artery ligation-induced heart failure through improving mitochondrial function via regulating ROS generation and CaMKII signaling pathways. *Frontiers in Pharmacology*.

[47] Chu J. X., Li G. M., Gao X. J., Wang J. X., Han S. Y. (2014). Buckwheat rutin inhibits AngII-induced cardiomyocyte hypertrophy via blockade of CaN-dependent signal pathway. *Iranian Journal of Pharmaceutical Research: IJPR*.

[48] Zhang M. X., Zheng J. K., Wang W. W. (2019). Exchange-protein activated by cAMP (EPAC) regulates L-type calcium channel in atrial fibrillation of heart failure model. *European Review for Medical and Pharmacological Sciences*.

[49] Neef S., Steffens A., Pellicena P. (2018). Improvement of cardiomyocyte function by a novel pyrimidine-based CaMKII-inhibitor. *Journal of Molecular and Cellular Cardiology*.

[50] Park W. J., Oh J. G. (2013). SERCA2a: a prime target for modulation of cardiac contractility during heart failure. *BMB Reports*.

[51] Olson E. N., Molkentin J. D. (1999). Prevention of cardiac hypertrophy by calcineurin inhibition: hope or hype?. *Circulation Research*.

